# Management of cardiopulmonary arrest in an educational video: contributions to education in pediatric nursing*

**DOI:** 10.1590/1518-8345.3680.3410

**Published:** 2021-04-12

**Authors:** Gabriel Zanin Sanguino, Maria Cândida de Carvalho Furtado, Simone de Godoy, Jéssica Batistela Vicente, Jacqueline Rodrigues da Silva

**Affiliations:** 1 Universidade de São Paulo, Escola de Enfermagem de Ribeirão Preto, PAHO/WHO Collaborating Centre for Nursing Research Development, Ribeirão Preto, SP, Brazil.; 2 Scholarship holder at the Conselho Nacional de Desenvolvimento Científico e Tecnológico (CNPq), Brazil.

**Keywords:** Instructional Films and Videos, Educational Technology, Nursing Education, Heart Arrest, Respiratory Insufficiency, Pediatrics, Filmes e Vídeos Educativos, Tecnologia Educacional, Educação em Enfermagem, Parada Cardíaca, Insuficiência Respiratória, Pediatria, Películas y Videos Educativos, Tecnología Educacional, Educación en Enfermería, Paro Cardíaco, Insuficiencia Respiratoria, Pediatría

## Abstract

**Objective:**

to make and validate an educational video about the management of pediatric cardiopulmonary arrest caused by respiratory failure.

**Method:**

methodological study developed in three stages: preparation and validation of a clinical case; production; and validation of educational video. To build the educational video, the Fleming, Reynolds and Wallace methodological framework was used.

**Results:**

the clinical case was validated by ten expert referees, who worked as nurses in different regions in Brazil, by completing an online form, with one round of evaluation and calculation of the content validity index. The educational video was validated by three expert judges and 25 nursing students in an evaluation round and contains six scenes, with duration of nine minutes and 56 seconds. An agreement over 80% was reached for most of the items in both the clinical case and the educational video.

**Conclusion:**

the educational video proved valid regarding face and content. This educational technology has the potential to meet the demands of students, who are digital natives, related to teaching of management of pediatric cardiopulmonary arrest caused by respiratory failure.

## Abstract (pt)

**Objetivo:**

construir e validar um vídeo educativo sobre o manejo da parada cardiorrespiratória pediátrica por insuficiência respiratória.

**Método:**

estudo metodológico desenvolvido em três etapas: elaboração e validação de um caso clínico; produção; e validação de vídeo educativo. Para construção do vídeo educativo foi utilizado o referencial metodológico de Fleming, Reynolds e Wallace.

**Resultados:**

o caso clínico foi validado por 10 juízes expertises e enfermeiros de diferentes regiões do Brasil, por meio de formulário *on-line*, com uma rodada de avaliação e cálculo do Índice de Validação de Conteúdo. O vídeo educativo foi validado por três juízes expertises e 25 acadêmicos de Enfermagem em uma rodada de avaliação e contém seis cenas e duração de nove minutos e cinquenta e seis segundos. Tanto o caso clínico quanto o vídeo educativo obteve-se para a maior parte dos itens, 80% ou mais de concordância.

**Conclusão:**

vídeo educativo mostrou-se válido quanto à aparência e ao conteúdo. Essa tecnologia educacional tem potencial para atender às demandas de alunos, nativos digitais, tanto quanto ao ensino do manejo da parada cardiorrespiratória pediátrica ocasionada por insuficiência respiratória.

## Abstract (es)

**Objetivo:**

construir y validar un video educativo sobre manejo del paro cardiorrespiratorio pediátrico por insuficiencia respiratoria.

**Método:**

estudio metodológico desarrollado en tres etapas: elaboración y validación de un caso clínico; producción; y validación de video educativo. Para la construcción del video educativo se utilizó el marco metodológico de Fleming, Reynolds y Wallace.

**Resultados:**

el caso clínico fue validado por 10 jurados expertos, enfermeros de diferentes regiones de Brasil, mediante formulario online, en una ronda de evaluación y calculándose Índice de Validación de Contenido. El video educativo fue validado por tres jueces expertos y 25 estudiantes de enfermería en una ronda de evaluación y contiene seis escenas, de nueve minutos cincuenta y seis segundos. Se obtuvo concordancia de 80% o superior para la mayoría de los ítems del caso clínico y del video educativo.

**Conclusión:**

el video educativo demostró validez en cuanto a apariencia y contenido. Esta tecnología educacional tiene potencial para satisfacer la demanda de alumnos nativos digitales en la enseñanza del manejo del paro cardiorrespiratorio pediátrico ocasionado por insuficiencia respiratoria.

## Introduction

Cardiopulmonary arrest (CPA) is a phenomenon described as the abrupt cessation of circulatory, respiratory, and cerebral activities, which directly interferes with blood circulation and efficacy of the pulmonary system to promote gas exchanges^(^ 1 ^)^.

In pediatrics, the main causes of CPA are shock and respiratory failure. This is defined as the respiratory system’s incapacity to keep its normal functions and effectively carry out the gas exchange mechanism^(^ 2 ^)^.

When the management of pediatric cardiopulmonary resuscitation is considered, preparation and technical and scientific knowledge of nurses is important to reverse the clinical condition shown and help patients get a better prognosis in this emergency situation^(^ 3 ^)^.

This preparation can be offered during training of these professionals over undergraduate courses. However, it is understood that it is necessary to take into account the students’ profile so the learning process is appealing and leads to the acquisition of the knowledge they are intended to have. There is widespread availability of contents on the internet, and authors identified that undergraduate students access knowledge by means of different learning methods, especially those involving technological resources, and have decreasing interest in classes given the traditional way^(^ 4 ^)^. Born in an era of great availability and access to digital media, these students are known as digital natives^(^ 5 ^)^.

The change in the profile of undergraduate students calls for the urgent development of educational materials that provide students with autonomy and raise their interest during the teaching-learning process so they can receive training that qualifies them for the job market, reduces the number of dropouts and gives the students who get a degree the possibility of developing their professional activities actively in face of the needs of the society to which they belong^(^ 6 ^)^.

To design the education of this population and follow technological progress, the universities’ teaching staff must use active and innovative methodologies so students can build their knowledge and feel like participating in teaching activities^(^ 7 ^)^.

The use of different active teaching approaches and methodologies has become an increasingly present and necessary practice in the nursing training sphere, especially those that incorporate technologies for the development of educational materials^(^ 7 ^)^.

Educational videos have stood out in the nursing field among the educational tools and methodologies that offer innovative and technological resources to allow students to learn. They are considered a low-cost, easily accessible educational technology that offers immediate behavioral changes^(^ 8 ^)^.

The development of educational videos relies on designing a material with a pedagogical basis and purpose. This material must also be validated to guarantee that its objectives are achieved^(^ 9 ^)^.

Because of the relevance of a meaningful learning, in which students are the main axis, that results in timely and proper care to children in critical health situation, the objective of the present study was to make and validate an educational video about the management of pediatric CPA caused by respiratory failure.

## Method

This was a methodological study whose data collection occurred between March and October 2018. It had three steps: elaboration and validation of a clinical case, making of an educational video, and evaluation of this video.

The methodological framework proposed by Fleming, Reynolds, and Wallace^(^ 10 ^)^ was applied to the making of the video.

The first step consisted of elaborating and validating a clinical case. It was developed based on experiences of the authors, clinical nurses and professors in the areas of pediatric nursing and development of educational technologies. Eleven expert referees were invited to participate in the evaluation of the video, and ten replied. Selection and inclusion of these professionals were carried out after analysis of their curriculum, available on the Lattes Platform. The search criteria used were being a nurse and filling out an adapted script^(^ 11 ^)^ that was designed to collect information on academic titles, clinical practice time, publications in the field, and participation in scientific events.

The second step referred to the making of the educational video, which was executed having the clinical case as a starting point. For the video evaluation, three expert referees and 59 students enrolled in a nursing undergraduate course at public higher education institution were invited. These students validated the video during their participation in an extension course offered by the authors. The inclusion criteria were being a nursing undergraduate student enrolled from the third semester of the course onward and fully filling out the study forms online. The students who did not meet the latter criterion were excluded. Twenty-five students completed the extension course.

The making of the video had three steps: pre-production, production, and post-production^(^ 10 ^)^.

During pre-production, a clinical case about pediatric CPA caused by respiratory failure was elaborated and validated. Subsequently, the expert referees received an on-line form, via Google Forms, containing free and informed consent forms and a characterization section whose objective was gathering sociodemographic data. To evaluate the clinical case, the content was considered paragraph *per* paragraph and, for each one, the referees issued a report related to the use of technical jargon, the vocabulary used, and clarity and reliability of the information. The referees provided answers by marking whether the assessed items were suitable or not and offered suggestions when they considered pertinent. At the end, they could dwell on their perceptions and considerations about the clinical case.

It is important to stress that the number of respondents was even (ten expert referees) and that there was no tie between agreement and disagreement in the evaluation of the items. Consequently, it was not necessary to add another expert referee to obtain the final definition about the content of the clinical case.

Evaluation of the clinical case construct was carried out by calculating the content validity index (CVI)^(^ 12 ^)^, which is the sum of the expert referees in positive agreement divided by the total number of expert referees. The method used was the scale-level CVI based on the average, which corresponds to the sum of CVIs divided by the total number of items. The established acceptance criterion was a CVI ≥ 0.8.

After validation, the clinical case was adapted into the video script format, with no addition or change in its content. The script included the description of human resources with their characterization (two nurses, two nursing technicians, and the mother), a numbered description of physical resources, and the description of six scenes with orientations related to contexts, camera moves, and lines of the characters.

After that, the clinical case was storyboarded, with the script being used as the source material to produce the video content. The video was filmed in an outpatient facility environment at a public higher education institution, with the presence of six actors and a high-fidelity child manikin.

In the post-production step, the recorded material was edited in cooperation with a multimedia material development team. This phase included insertion of visual elements, such as figures and texts related to the illustrations. Six scenes made up the video. After completion of the video production, the material was submitted to evaluation by three expert referees. Subsequently, the video was inserted into a virtual learning environment to be validated by the 25 nursing undergraduate students. They fully filled out a form designed as a Likert scale with five possible scores, each associated with the following statements: I totally agree (5 points), I agree (4 points), I neither agree nor disagree (3 points), I disagree (2 points), and I totally disagree (1 point). These statements were used to evaluate the functioning, usability, efficiency, environment, and audiovisual resources of the produced video.

The interface validation form of the educational video was adapted from studies^(^ 13 ^-^ 14 ^)^ that have developed and used forms with the Likert scale structure as a tool to validate face in educational videos.

The final step was a descriptive data analysis. The answers were systematized using tabulation of the gathered information in a Microsoft Excel spreadsheet.

The present study was approved by the Undergraduate Studies Committee at the educational institution and by the research ethics committee as *per* Certificate of Presentation for Ethical Evaluation no. 84077418.3.0000.5393 and report no. 2,596,505. All the referees, authors, and undergraduate students read the form and accepted to participate in the study. The actors signed an authorization form allowing image use.

## Results

The first step, the elaboration of the video, resulted in a script and a storyboard with validated content that originated a video version with six scenes, with a total duration of nine minutes and 56 seconds.

The ten expert referees that participated in the content validation of the clinical case were women and nurses, had an average age of 38.4 years, and an average time of 16.5 years since graduation. Regarding their origin, three were from the Brazilian state of Paraná and three from the Brazilian state of São Paulo, and the Brazilian states of Santa Catarina, Paraíba, and Goiás and the Federal District contributed with one nurse each.

During the study execution period, seven referees worked as professors, two were nurses in hospitals that were part of public universities, and one had no employment bond. The average time working on their current job was six years.

For validation, the clinical case was split into nine paragraphs and 36 questions, which covered care to the child and their mother since arrival at the health service until completion of care actions oriented toward the recovery of the child after CPA caused by respiratory failure. Nine questions reached the maximum CVI value of 1.0, 14 obtained a CVI of 0.9, and 13 got a CVI of 0.8.

Information clarity, information reliability, and vocabulary reached a CVI of 0.9. The item technical jargon obtained a CVI of 0.86. The overall CVI of the clinical case was 0.89.

There were suggestions for the clinical case content. They addressed adjustments in the sentences to achieve greater information clarity, and all of them were accepted. The third, fourth, and six paragraphs of the clinical case were those that obtained the highest number of suggestions for adjustments that could increase the reliability of the data shown and the ease to understand the clinical case.

The validated clinical case was adapted into the script and storyboard formats, which were the basis for production of the educational video.

**Figure 1:**
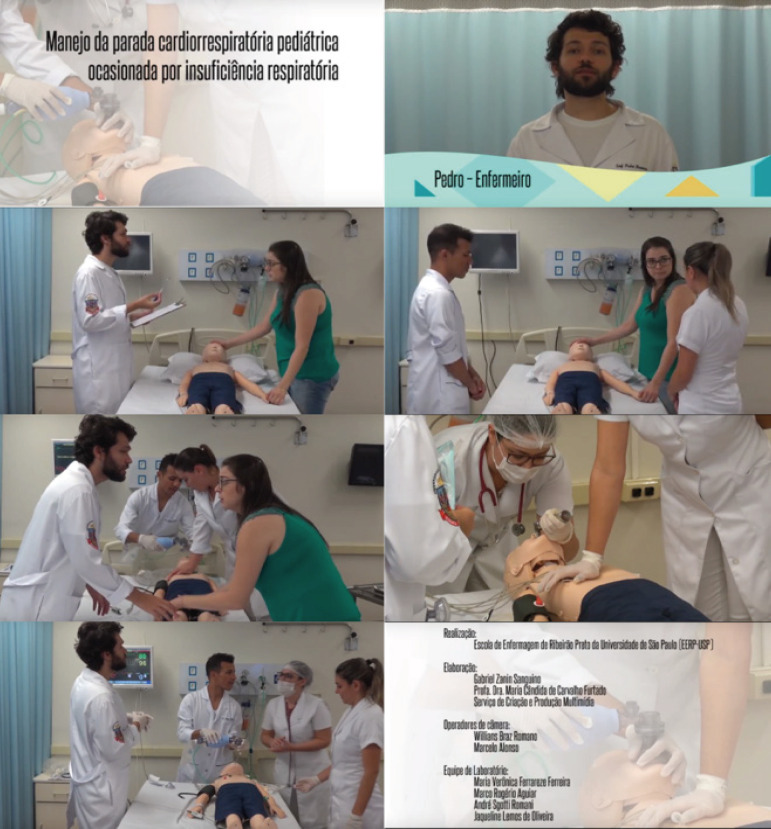
Edited scenes of the educational video Management of pediatric cardiopulmonary arrest caused by respiratory failure; Pedro - Nurse; Production: Ribeirão Preto College of Nursing at the University of São Paulo; Creation: Gabriel Zanin Sanguino, Professor Maria Cândida de Carvalho Furtado, Ph.D., Multimidia Production and Creation Section; Camera operators: Willians Braz Romano, Marcelo Alonso; Laboratory team: Maria Verônica Ferrareze Ferreira, Marco Rogério Aguiar, André Sgotii Romani, Jaqueline Lemos de Oliveira

*Source: authors’ image right

The final produced video had a title, opening, scenes showing the care delivered to the child with CPA caused by respiratory failure, conclusion, and credits. These characteristics can be seen in Figure 1.

After the shooting, three referees validated the educational video (two nurses that participated in the validation of the clinical case and one communications analyst). Two were women and one was a man, and their ages and time since graduation ranged from 29 to 51 years and seven to 27 years, respectively. They all worked in higher education public institutions, two in the state of São Paulo and one in the state of Paraná. Their time working in the current job varied between 11 months and 31 years.

Functionality, usability, efficiency, environment, and audiovisual resources were the core topics in the set of statements designed to validate the video. The authors opted to adapt forms used by other researchers^(^ 13 ^-^ 14 ^)^, and the statements were presented with five Likert scale-like answers: I totally agree (5 points), I agree (4 points), I neither agree nor disagree (3 points), I disagree (2 points), and I totally disagree (1 point).

The statements related to the functionality and usability categories obtained the answers “I totally agree” or “I agree”. Efficiency and environment received each a “I neither agree nor disagree” statement regarding the distribution of information in the screen and lighting, respectively.

In the audiovisual resources category, one referee neither agreed nor disagreed on two statements that mentioned clarity and the tone of voice of the actors in the video and the possibility of going back to previous scenes when desired.

All the other statements in the validation script obtained “I totally agree” or “I agree” answers.

The validation proposed to the nursing undergraduate students (the video as an educational tool) had the participation of 13 (52%) students from the nursing undergraduate course and 12 (48%) from the nursing undergraduate course that gives a license to develop teaching activities. Most of the students (23 or 92%) were younger than 25 years and had had the care to hospitalized child subject addressed already, which occurred in the third year of the course (14 students or 56%). When asked whether they had already used some sort of educational technology, 21 (84%) confirmed that experience. Using the internet to search contents as a support to the training they had in the undergraduate course was unanimity in the examined sample.

The reasons why 34 nursing undergraduate students did not complete their participation in the extension course were not verified in the present study. However, it can be stated that their exclusion did not impact the validation process of the educational material.

The students had access to the video for 30 consecutive days, and they could watch it any time and as many times as they wanted. When this 30-day period was over, they were asked to fill out the video validation script.

Regarding the educational video validation aspects, the answers given by the students are shown in Table 1. In accordance with the opinion of the referees, most students evaluated the video positively. A small percentage chose the option “I neither agree nor disagree” for 11 items. No student picked the options “I disagree” and/or “I totally disagree”.

**Table 1:** Distribution of nursing undergraduate students' answers (n=25) related to the educational video according to agreement levels. The items of the validation instrument are listed in the first column. Ribeirão Preto, SP, Brazil, 2018

	I totally agree		I agree		I neither agree nor disagree	
	N	%	n	%	n	%
Functionality						
The video is a tool suitable to the objective it is intended to.	19	76	6	24	-	-
The video allows to generate positive results in the teaching-learning process about pediatric CPA*.	6	60	10	40	-	-
Usability						
The video is easy to use.	18	72	5	20	2	8
It is easy to learn how care to children with CPA* caused by respiratory failure is provided.	3	12	17	68	4	16
The video allows users to easily apply the explored concepts to hospital practice.	7	28	15	60	1	4
The video duration is adequate for users to have greater closeness with its content.	14	56	8	32	2	8
The video makes the learning process easier.	18	72	7	28	-	-
Efficiency						
The video follows a logical sequence.	17	68	8	32	-	-
The information is properly distributed over the screen from the spatial point of view.	15	60	8	32	1	4
The video refers to a situation that occurs in the hospital setting.	12	28	11	44	1	4
Environment						
The laboratory setting did not interfere with the accuracy of the CPA* care.	9	36	10	40	4	16
The lighting is adequate to watch the scenes.	14	56	10	40	1	4
Audiovisual resources						
The dialogues in the video are delivered efficiently and intelligibly.	12	48	9	36	3	12
The voice of the actors is clear and its level is adequate.	14	56	8	32	2	8
The number and characterization of characters meet the proposed objective.	15	60	9	36	1	4
It is possible to go back to any part of any scene when desired.	19	76	6	24	-	-
The number of scenes is compatible with the video duration.	17	68	8	32	-	-

* CPA = Cardiopulmonary arrest

From these results, it was concluded that the video interface was validated as a material with the potential to contribute to training on the examined subject in the opinion of the consulted expert referees and students.

## Discussion

The teaching of pediatric emergencies, and specifically of the management of pediatric CPA, has important gaps for nursing professionals related to the theoretical and scientific knowledge to efficiently work in face of this emergency^(^ 15 ^-^ 16 ^)^.

The literature addresses CPA approach and use of active and/or educational methodologies to be applied to health professionals with studies on the use of these tools to teach the subject to medical students^(^ 17 ^-^ 18 ^)^, hence the importance of the present study, which is focused on the learning process of nursing students.

It is understood that, with the change in the profile of students at higher education institutions, educational approaches must meet their needs and expectations. The production of multimedia material is a tool that strengthens the teaching-learning process^(^ 19 ^)^.

The use of digital information and communications technologies has become increasingly common in the academic universe, especially in the undergraduate level, and the training of nurses has stood out in this scenario. A study^(^ 19 ^)^ showed that applying this type of technology positively impacts the population’s health.

The development of educational videos is a practice that can contribute to transforming knowledge. This type of material leads to autonomy and independence, empowering students during the process of getting knowledge about varied subjects^(^ 8 ^)^.

Educational videos, an example of active technological methodologies, are digital tools that correspond to the recording of images and sounds with posterior reproduction and have the potential to stimulate the people who watch them and hold their attention^(^ 13 ^)^.

With an increasing prominence in the academic context, the production of educational videos has been a research subject whose growing importance in the nursing area is expressed by the existence of studies that have addressed the development and validation of this type of material to help in the learning process of several subjects^(^ 14 ^,^ 20 ^)^.

It is important to emphasize the relevance of using a methodological framework^(^ 10 ^)^ in the process of creating the material that will substantiate the educational video, a practice carried out by the authors of the present study^(^ 21 ^-^ 22 ^)^.

The elaboration of a clinical case for validation before the preparation of the storyboard and the writing of the script was an approach different from that used in a similar study^(^ 14 ^)^. This characteristic is explained by the fact that the present study is part of a more comprehensive research project that concomitantly investigates other educational approaches. The clinical case was a guiding material for other learning tools too.

It was identified that making the video script available in the validation by expert referees was a fundamental element in the development of an educational video. However, the approach used in the present study (proposing a clinical case that, after validation, was adapted into the script format) allowed a higher accuracy in the preparation of the material used to create the scenes to be filmed.

The educational video development process was facilitated by the conditions offered by the educational institution to which the study was linked. It is understood that the availability of physical and human resources for the creation of this type of material is fundamental for making a satisfactory product^(^ 23 ^)^. It is important to emphasize that support from educational institutions to develop materials such as that described in the present study motivates and encourages professionals to carry out that practice more often^(^ 19 ^)^.

The result is in agreement with the recommendation that the duration of educational videos does not exceed 15 minutes^(^ 24 ^)^, which has been followed in other studies^(^ 14 ^,^ 22 ^,^ 25 ^)^.

Using well-defined criteria to select referees is an indispensable practice and directly impacted the development of the present study^(^ 26 ^)^. It must be stressed that two referees participated in the validation of both the clinical case and the video, which allowed to monitor the developed material and provided greater clarity in the steps for the elaboration of the final product.

Making the material available for 30 days in the process of evaluation by nursing undergraduate students allowed them to access the video as many times as they considered necessary to fill out the validation form on-line subsequently.

The educational video validation process carried out by referees and nursing undergraduate students was satisfactory, since there was a predominance of positive evaluations for the examined material.

Production of videos and their use for academic purposes allow a greater engagement of students in the teaching-learning process, encourages them to develop their autonomy, and have the potential to immediately transform knowledge^(^ 8 ^)^.

In the training of nursing professionals, the use of digital technologies can be one of the means to provide this workforce with resources and decisively meet the demands that emerge in an increasingly digital world^(^ 27 ^)^. The author of the mentioned study emphasizes that the maturity of the digital transformation in the health area is an opportunity to qualify the nursing work and points to the progress in skills inherent in the digital field as a challenge to this health area^(^ 27 ^)^. It is understood that the present study showed that this path can be initiated during the training of future professionals.

A limitation of the study was the fact that the questions involving the changes in the knowledge of nursing undergraduate students were not deeply examined. The focus of the investigation was the face and content of the produced material.

## Conclusion

An educational video on pediatric CPA caused by respiratory failure was made and validated by expert referees and nursing undergraduate students. It was considered that the final version of the educational material is fit to be used with students in nursing undergraduate courses. The present study contributed to the area by making an innovative digital tool available to teaching practices about the management of pediatric CPA caused by respiratory failure oriented toward nursing undergraduate students.

In a context with constant technological evolution and academic needs that promote the building of knowledge according to the demands of the students, the scientific offer of methodological aspects related to the development and validation of technological and digital learning objects encourages an education compatible with the current academic reality.
